# 1p36 Deletion Syndrome and Left Ventricular Non-compaction Cardiomyopathy—Two Cases Report

**DOI:** 10.3389/fped.2021.653633

**Published:** 2021-06-07

**Authors:** Subin Jang, Allison Taber, Michael G. Bateman, Marie E. Steiner, Rebecca K. Ameduri, Massimo Griselli

**Affiliations:** ^1^Division of Pediatric Cardiac Surgery, Department of Surgery, University of Minnesota Masonic Children's Hospital, Minneapolis, MN, United States; ^2^Division of Pediatric Critical Care, Department of Pediatrics, University of Minnesota Masonic Children's Hospital, Minneapolis, MN, United States; ^3^Department of Surgery, University of Minnesota Masonic Children's Hospital, Minneapolis, MN, United States; ^4^Division of Pediatric Cardiology, Department of Pediatrics, University of Minnesota Masonic Children's Hospital, Minneapolis, MN, United States

**Keywords:** 1p36 deletion syndrome, LVNC, non-compaction, case report, cardiomyopathy

## Abstract

1p36 deletion is the most common terminal deletion syndrome in humans. Herein, we report two cases, a 5-month-old female and a 14.5-year-old female, both with 1p36 deletion and left ventricular non-compaction cardiomyopathy. They presented with severely depressed left ventricle function and underwent heart transplantation with excellent outcomes. Given the incidence of heart defects and cardiomyopathy in 1p36 deletion syndrome, it should be recommended that children with this genetic condition have screening for cardiac disease. These cases add to the current literature by demonstrating the potential therapeutic options for non-compaction in 1p36 deletion syndrome and showed the favorable outcomes.

## Introduction

The 1p36 deletion syndrome is the most common terminal deletion syndrome in humans, which occurs in 1 in 5,000 live births (1). It is associated with many clinical features, including developmental delay, intellectual disability, auditory and ophthalmologic impairment, and heart defects including cardiomyopathy (2, 3).

Previously, there have been no reports of outcomes following cardiac transplantation in children with 1p36 deletion syndrome. This paper presents two cases of patients with 1p36 deletion and left-ventricular non-compaction (LVNC) cardiomyopathy. Case 1 was a 5-month-old girl who underwent repair of congenital heart defect, followed by mechanical circulatory support and then ultimately received a heart transplantation. Case 2 was a 14.5-year-old patient who underwent heart transplantation with great post-transplant outcome.

These cases add to the current knowledge of 1p36 deletion syndrome. They demonstrated the favorable outcomes following heart transplantation. Although the experience is limited to these two cases, neither have had rejection or significant cardiac complications.

## Case Description And Diagnostic Assessment

### Patient 1

A 5-month-old, 6.1-kg girl with 1p36 deletion syndrome with acute-on-chronic left ventricular failure due to LVNC cardiomyopathy was admitted to our institution for worsening heart failure and possible need for cardiac transplantation. She had a history of hypotonia, global developmental delay, and feeding intolerance. There was no known 1p36 deletion family history, although her mother has cerebral palsy, developmental delays, spastic dysplasia, history of traumatic brain injury, and Ehlers Danlos syndrome with cardiac diagnosis of mitral valve prolapse and dilated aorta. Proband was born full-term and passed the newborn Critical Congenital Heart Disease (CCHD) screen, however showed poor color and poor feedings. A low oxygen saturation led to an urgent echocardiogram. Coarctation of the aorta was diagnosed shortly after birth with depressed left-ventricular function, and repair with an end-to-end anastomosis at 19 days of age was undertaken without improvement in ventricular function. The echocardiogram demonstrated a left-ventricular ejection fraction (EF) of 28% after a couple of days and 17% after a month from the CoA procedure. Further investigation led to the diagnosis of LVNC and 1p36 deletion.

**Figure 1:**
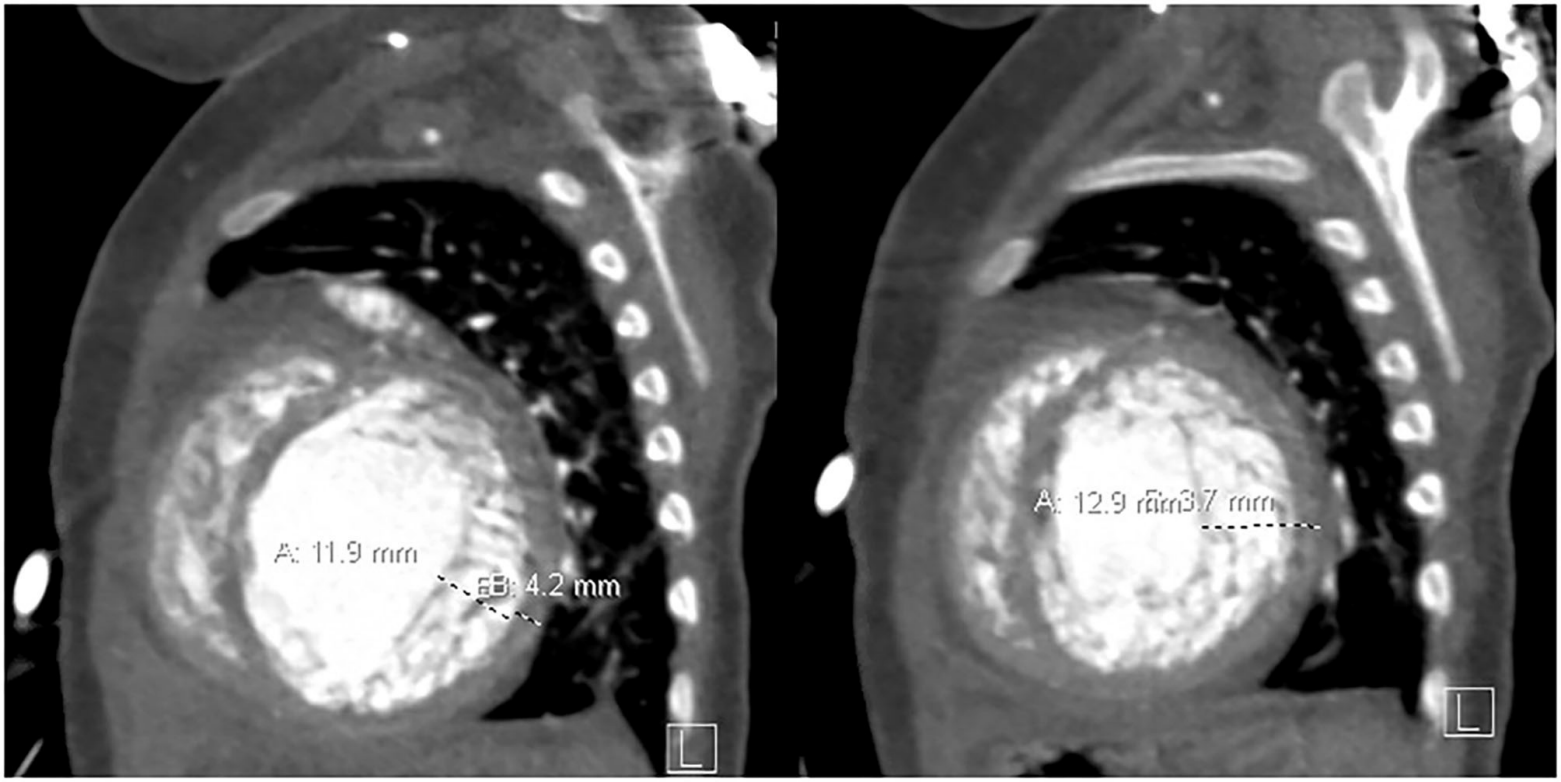
Non-compacted myocardium measures 11.9–12.9 mm, and the compacted myocardium measures 3.7–4.2 mm in the sagittal plane.

After initial improvement with medical treatment at an outside institution, she was admitted to our hospital with deteriorating clinical status for more aggressive in-hospital heart failure management including mechanical circulatory support (MCS). Imaging at the time of admission showed LVNC with trabeculated myocardium measuring 11.9–12.9 mm in thickness with prominent trabeculations and deep intertrabecular recesses communicating with the ventricular cavity (Figure 1). There was marked left-ventricular enlargement and decreased systolic function with an EF of 22%. After evaluation, the patient was listed for cardiac transplantation.

**Figure 2:**
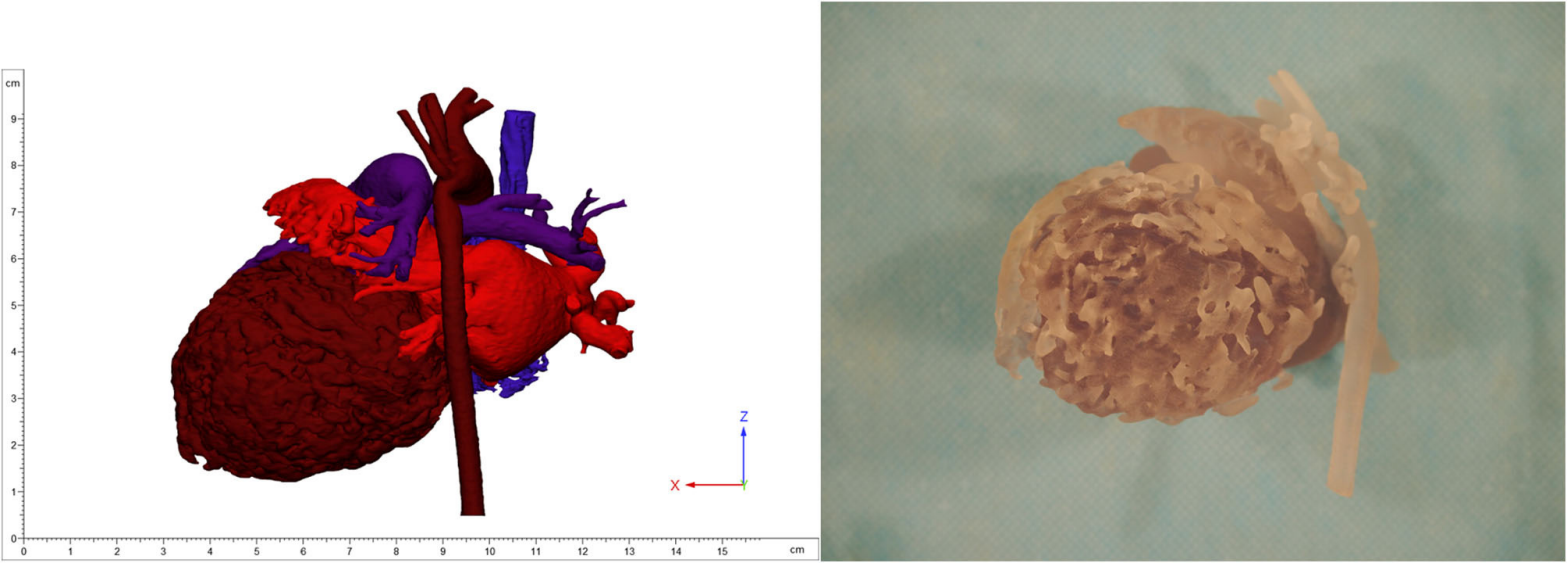
3D image (left) and printing (right) of non-compaction cardiomyopathy.

Despite optimization of medical therapy, she required MCS with peripheral veno-arterial extracorporeal membrane oxygenation (VA-ECMO) for stabilization, followed by left-ventricular assist device (LVAD) Berlin Heart Excor (BHE) implantation. Anticipating the patient's need for MCS, 3D models of the heart were produced locally utilizing contrast enhanced computed tomography (CT) images to aid in surgical planning (Figure 2). Ultimately, a 6-mm inflow left-ventricular cannula and 6-mm arterial outflow cannula with 8-mm Dacron tube graft were used for the LVAD and connected to a 15-ml BHE pump. Extensive trabeculation resection was undertaken through the left-ventricular apex to achieve clearance for the inflow cannula. A successful aortic reconstruction to relieve residual coarctation was also performed at the time of LVAD implantation. An immediate post-operative echocardiogram revealed adequate decompression of the left ventricle. Fortunately, a suitable organ donor became available 7 days after BHE implantation, and the patient was successfully bridged to orthotopic heart transplantation.

As of this writing, the patient is now 2 years 5 months after heart transplantation and has done well from cardiac perspective, with normal ventricular function and no rejection history (**Figure 4**).

### Patient 2

A 14.5-year-old, 87.5-kg female with 1p36 deletion syndrome and associated developmental delays presented in cardiorespiratory failure. Upon admission to our institution, she was diagnosed with LVNC cardiomyopathy with severely depressed left ventricle function. She had a history of low muscle tone and some congenital hypotonia, feeding difficulties, mild facial dysmorphism, underlying gross and global developmental delay, and partial hearing loss. There was no known 1p36 deletion family history.

She had been in her usual state of health without cardiac issues until 1 week prior to presentation when she developed persistent abdominal pain, worsening appetite, and orthopnea. She presented to local emergency department, and given sepsis concern, she was transferred to an acute hospital where she was intubated and started on dopamine and milrinone due to EF of 23%. She was then transferred to our institution for further stabilization of her heart failure as well as evaluation for cardiac transplantation or LVAD.

**Figure 3:**
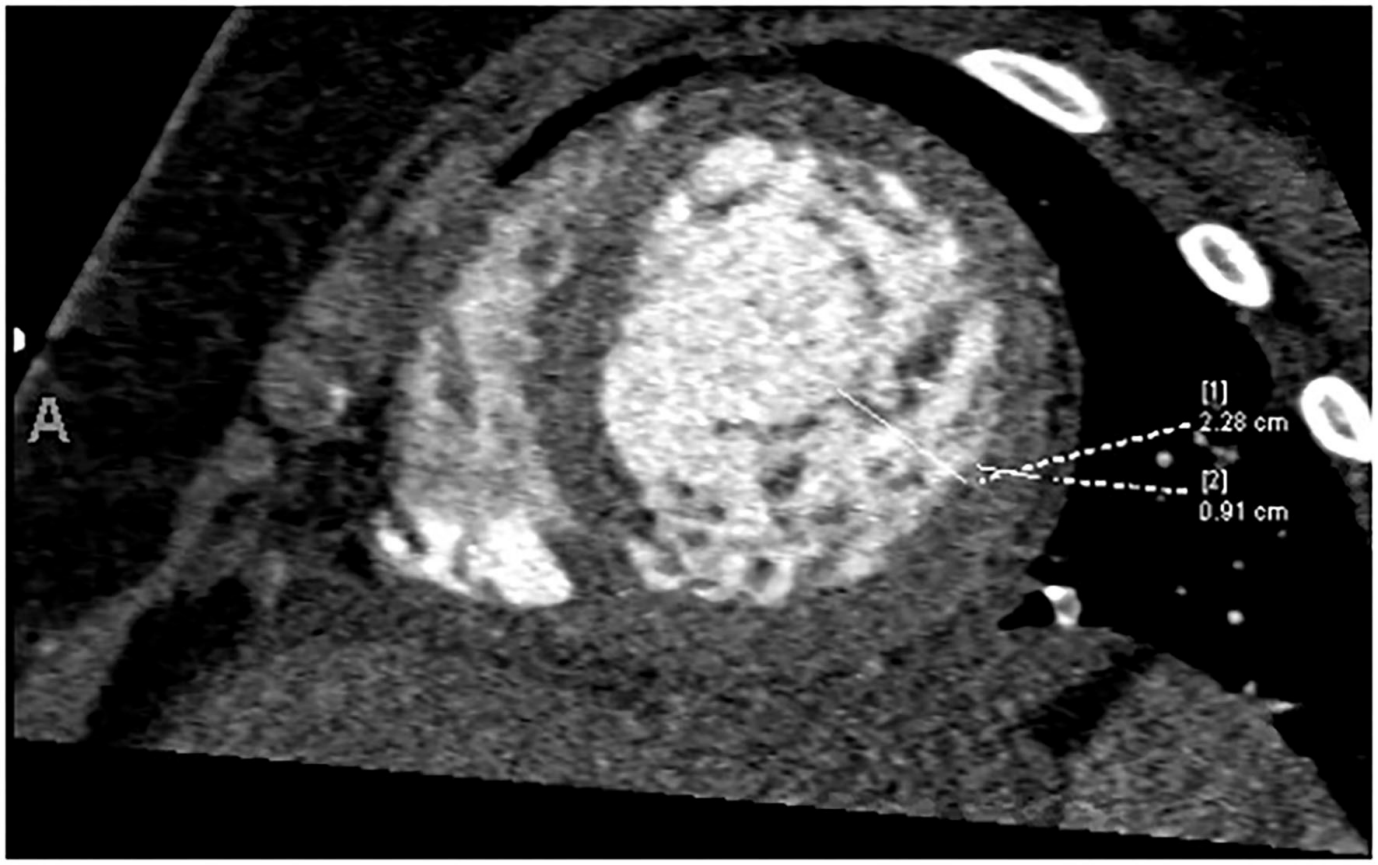
Increased trabeculation in the left ventricle, particularly in the midsections and apex with a non-compacted to compacted ratio of 2.5.

The echocardiogram at admission showed LVNC cardiomyopathy with decreased left-ventricular systolic function with EF of 34%. The patient was found to be in ventricular tachycardia with hemodynamic compromise, likely secondary to heart failure and volume overload. The arrhythmia was resolved with pleural effusion drainage and weaning of inotropic support, and it did not recur prior to transplantation. Computed tomography angiography (CTA) showed increased trabeculations in the left ventricle, particularly in the midsections and apex with a non-compacted to compacted ratio of 2.5 (Figure 3). Extubation was attempted, but ultimately, she failed due to poor cardiac function. Right heart catheterization revealed favorable Pulmonary vascular resistance (PVR), while on inotropic drips. The patient was listed for cardiac transplantation, and 2 days later, a suitable organ donor became available, and the patient was successfully transplanted.

**Figure 4:**
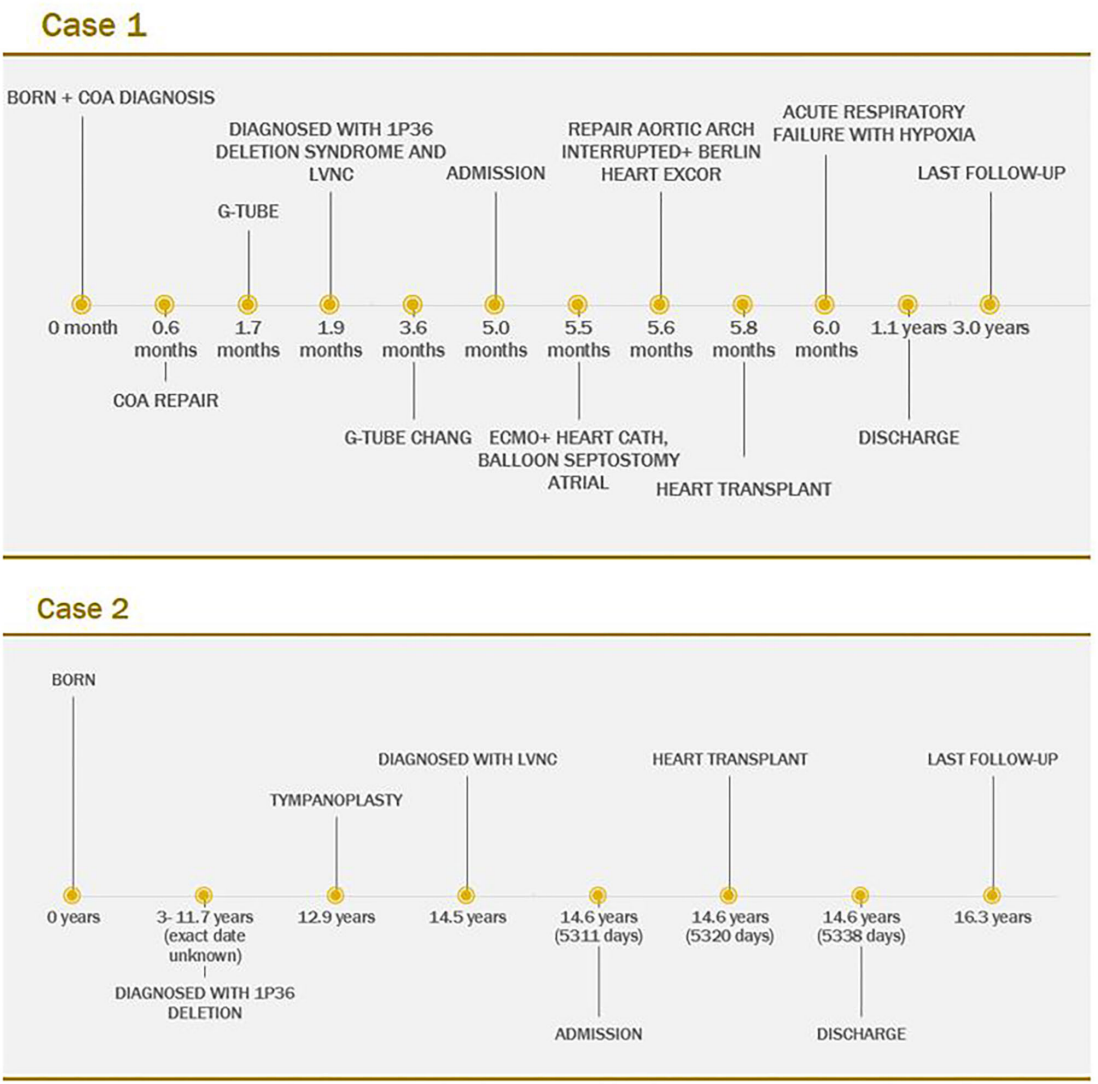
Timeline of Case 1 and Case 2.

As of this writing, the patient is now 1 year 8 months after cardiac transplantation and has done well from a cardiac perspective with normal function and no history of rejection (Figure 4).

## Discussion

1p36 deletion syndrome is a common subtelomeric microdeletion syndrome (3). Most of the deletion occurs in the maternally derived chromosome (1). The main characteristics of 1p36 deletion syndrome includes intellectual disability, growth delay, seizure, vision and hearing impairment, and craniofacial dysmorphism. The common facial appearance features are straight eyebrows, microbrachycephaly, deep-set eyes, flat nasal bridge, pointed-chin, and low-set ears (1, 3). There are limited data on natural progression and long-term outcomes in patients with 1p36 deletion (4).

Non-compaction cardiomyopathy is a distinct form of myocardial disorder, characterized by a two-layered ventricular wall with prominent trabeculations and deep, inter-trabecular recesses communicating with the ventricular cavity (5). This diagnosis may lead to heart failure, life-threatening arrhythmias, sudden death, or stroke, and mechanical circulatory devices may be used to assist the left ventricle as a bridge to transplantation (6).

LVNC is shown to be associated with many genes including 1p36 (7). Battaglia et al. reviewed 60 patients with 1p36 deletion syndrome, and 34 (71%) of 48 patients with sufficient data had congenital heart defects (3). Thirteen (27%) patients had cardiomyopathy, and 11 (23%) were the non-compaction type (3).

LVNC diagnosis might be underreported in 1p36 deletion syndrome: (1) when they have not been detected due to technical challenge, (2) if mild LVNC was evaluated as normal, or (3) because of only a subset of mutation carrier is affected among many other genes that are associated with LVNC (8). For our case 1, initially the depressed left-ventricular function was thought to be secondary to critical coarctation; however, when function did not improve post-operatively, the outside institution did further evaluation and diagnosed LVNC. Given the incidence of heart defects and cardiomyopathy in 1p36 deletion syndrome, it should be recommended that children with this genetic condition have screening echocardiograms.

We presented our two cases with the diagnosis of LVNC cardiomyopathy associated with 1p36 deletion syndrome who both successfully underwent heart transplantation and are doing well following transplant, although the follow-up period is relatively short. Our cases showed some of the 1p36 deletion characteristics that were described in current literature, including developmental delay, hypotonia, feeding difficulties, facial dysmorphism, and partial hearing loss. These cases contribute to the medical literature in evidence of cardiac anomalies associated with 1p36 deletion syndrome and support that cardiac transplantation is a viable option in patients with this combination of defects.

## Data Availability Statement

The original contributions presented in the study are included in the article/supplementary material, further inquiries can be directed to the corresponding author.

## Ethics Statement

The IRB determined that the proposed activity is not research involving human subjects as defined by DHHS and FDA regulations. Authors have obtained written informed consent from the patients and patients' families for the publication.

## Author Contributions

SJ, AT, and MB wrote the first draft manuscript with support from MG, MS, and RA. MG conceived of presenting these cases and supervised the project. SJ, MG, RA, and MS contributed to the final version of the manuscript. All authors provided critical feedback and helped shape the report.

## Conflict of Interest

The authors declare that the research was conducted in the absence of any commercial or financial relationships that could be construed as a potential conflict of interest.
